# Comorbid Influences on Generic Health-Related Quality of Life in COPD: A Systematic Review

**DOI:** 10.1371/journal.pone.0132670

**Published:** 2015-07-13

**Authors:** Manuel B. Huber, Margarethe E. Wacker, Claus F. Vogelmeier, Reiner Leidl

**Affiliations:** 1 Institute of Health Economics and Health Care Management, Helmholtz Zentrum München, German Research Center for Environmental Health (GmbH), Comprehensive Pneumology Center Munich (CPC-M), Member of the German Center for Lung Research (DZL), Neuherberg, Germany; 2 Department of Medicine, Pulmonary and Critical Care Medicine, Philipps-Universität Marburg, University Medical Centre Giessen and Marburg (UGMLC), Member of the German Center for Lung Research (DZL), Marburg, Germany; 3 Munich Center of Health Sciences, Ludwig-Maximilians-Universität, Munich, Germany; Pulmonary Research Institute at LungClinic Grosshansdorf, GERMANY

## Abstract

**Background:**

Chronic obstructive pulmonary disease (COPD) is a leading cause of mortality and of loss of disability-adjusted life years worldwide. It often is accompanied by the presence of comorbidity.

**Objectives:**

To systematically review the influence of COPD comorbidity on generic health-related quality of life (HRQoL).

**Methods:**

A systematic review approach was used to search the databases Pubmed, Embase and Cochrane Library for studies evaluating the influence of comorbidity on HRQoL in COPD. Identified studies were analyzed according to study characteristics, generic HRQoL measurement instrument, COPD severity and comorbid HRQoL impact. Studies using only non-generic instruments were excluded.

**Results:**

25 studies met the selection criteria. Seven studies utilized the EQ-5D, six studies each used the SF-36 or SF-12. The remaining studies used one of six other instruments each. Utilities were calculated by four EQ-5D studies and one 15D study. Patient populations covered both early and advanced stages of COPD and ranged from populations with mostly stage 1 and 2 to studies with patients classified mainly stage 3 and 4. Evidence was mainly created for cardiovascular disease, depression and anxiety as well as diabetes but also for quantitative comorbid associations. Strong evidence is pointing towards the significant negative association of depression and anxiety on reduced HRQoL in COPD patients. While all studies found the occurrence of specific comorbidities to decrease HRQoL in COPD patients, the orders of magnitude diverged. Due to different patient populations, different measurement tools and different concomitant diseases the study heterogeneity was high.

**Conclusions:**

Facilitating multimorbid intervention guidance, instead of applying a parsimony based single disease paradigm, should constitute an important goal for improving HRQoL of COPD patients in research and in clinical practice.

## Background

Generating over 76 million disability adjusted life-years (DALYs) globally in 2010, chronic obstructive pulmonary disease (COPD) surpassed road-traffic injuries, and when focusing on the US alone, it was the second highest contributor of DALYs after ischemic heart disease [1]. Smoking is the main risk factor for developing COPD [2], while all hallmarks of ageing [3] seem to influence its progression [4]. The pathogenesis of COPD is multidimensional [5, 6]. Inflammation, airway remodeling and fibrosis as well as tissue destruction seem to play constituting roles for the usually progressive nature of the disease [7]. There is currently no cure for COPD and the need for disease modifying treatments is still unmet [8] although possible targets seem promising [9, 10]. Comorbidity is common for COPD [11] and COPD patients use disproportional amounts of health services for the treatment of their comorbid conditions [12–16]. However, comorbidities are often considered as exclusion criteria for participants of COPD studies or are disregarded in respective evaluations [17]. In a study [18] from Italy in 2014, around 80% of COPD patients were treated by protocols derived from randomized clinical trials, for which they would not have been eligible to participate in. By doing so, a reductionist paradigm regarding disease classification [19, 20], largely based on end-stage appearance of symptoms, is compounded by strict study eligibility criteria. Taken together, this likely fails to account for different patho-phenotypes and thus mirrors a partial failure to reflect clinical reality. Health-related quality of life (HRQoL), next to survival and costs, is one important measure for cost-effectiveness of interventions. However, controversies about clinical implementation of HRQoL are still present [21]. The importance of improving clinical management and thus HRQoL of COPD patients afflicted by comorbidity has been object of investigation by several studies [22–25]. Therefore, evaluating comorbid influences on HRQoL in COPD could help to unravel disease constellations of interest for patients, physicians and payers. The aim of this review is to aggregate and summarize evidence for the influence of comorbidities on generic HRQoL in COPD.

## Methods

### Measuring HRQoL

HRQoL can be measured by different instruments. Some of these instruments are disease specific (e.g. St. George's Respiratory Questionnaire (SGRQ) [26]), while others are generic, meaning they can be compared among different fields of indication. Disease-specific instruments were excluded from this review. The rationale for this decision is rooted in the fact that disease-specific instruments were not designed to evaluate comorbid influences unless the effects are expressed by the index disease and can therefore be measured by the disease-specific instrument. For example, the widely used SGRQ was designed to measure the effects of airflow limitation on HRQoL [26] but it was not intended to measure effects unrelated to airflow limitation. In contrast, generic instruments were designed to measure HRQoL irrespective of multimorbidity and way of expression. Still, it could be interesting to evaluate the effect of different comorbidities on various measures of disease-specific outcome. This is beyond the scope of this review, however. Examples for well accepted and widely used generic instruments include the EuroQol five-dimension questionnaire (EQ-5D) [27], which, inter alia, was used in major COPD studies like the TOwards a Revolution in COPD Health (TORCH)-trial [28]. The EQ-5D consists of 5 descriptive questions (self-classifier) and a valuation by a visual analogue scale (VAS) labelled EQ-5D-VAS score. The results for the 5 dimensions can be transformed into utilities, which are needed for cost-effectiveness analysis. In order to derive population based utilities for different health states the most widely used method is time-trade-off (TTO) [29], typically surveyed in representative samples of the general population. The TTO procedure elicits the time in perfect health which respondents consider equal to a given time in a health state, with the relation of both rendering the health state’s value. Other important generic instruments include but are not limited to the 36-Item Short Form Health Survey (SF-36) [30], the 12-Item Short Form Health Survey (SF-12) [31], two instruments with pre-defined summary scores such as average across the items of one dimension, and the 15D questionnaire (15D) [32]. The SF-36 is made up of 36 items, which are grouped into 8 subdomains. For each subdomain, a score between 0 (worst) and 100 (best) can be reached. The SF-12 is a short version of the SF-36 and contains 12 items. These 12 items reproduce at least 90% variance of the physical component summary score (PCS) and the mental component summary score (MCS) from SF-36 [31]. The 15D is a 15-dimensional self-administered generic instrument which can be used a single and profile index score measure, also rendering utility measurement. Citations for other, less used instruments are provided in this review and can be used to gather more respective information. Beyond clinical assessment, utility measures of HRQoL provide a key effect measure in economic evaluation studies.

### Search strategy and exclusion criteria

The respective literature search was performed on May 5^th^ 2015. Studies only using disease-specific instruments were excluded. The publication date was not restricted. Pubmed was searched using the following terms: (((((copd[MeSH Terms]) OR copd) OR obstructive lung disease) OR obstructive pulmonary disease)) AND (((("Quality of Life"[Mesh]) OR quality of life) OR health status) OR "Health Status"[Mesh]) AND (("Comorbidity"[Mesh]) OR comorbid*)

**Fig 1:**
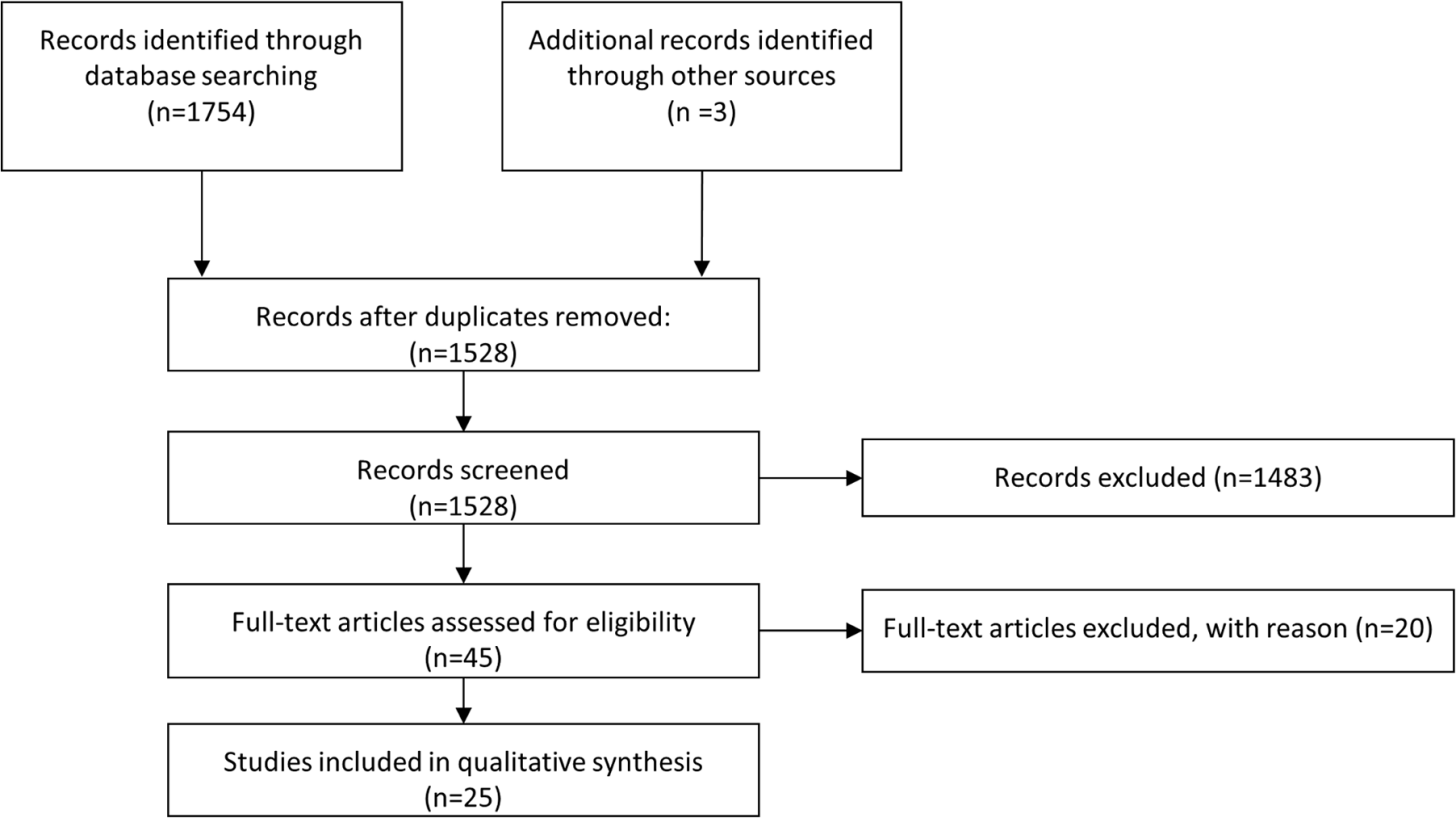
Study selection process.

This resulted in 1125 hits. Embase was searched by ((copd OR obstructive lung disease OR obstructive pulmonary disease) AND comorbid* AND (quality of life OR health status)) NOT SU = MEDLINE and was restricted for journal articles. 629 results were found. 3 records [33–35] were identified by hand search. In addition to this, the Cochrane Library was searched for respective reviews (COPD AND comorbid* AND quality of life) but none were found. Combining the results lead to 1757 studies in total. After removing duplicates, 1528 studies remained. The language filter (English, German) was implemented and studies using non-generic HRQoL instruments were removed. Studies that did not deliver comorbid based results, were also excluded. The PRISMA flow diagram [36] was used to depict the study selection process (Fig 1). The PRISMA checklist is annexed as supplementary data (see S1 File.) as well as a list (see S2 File.) with studies and their respective reason for exclusion.

### Data extraction

Basic population characteristics, generic HRQoL measurement instrument, severity of COPD, prevalence of comorbidities and the comorbid association regarding generic HRQoL were extracted from all selected studies under review. Valuation methods for index instruments like the EQ-5D and the 15D instrument were actively investigated for unless clearly stated in the respective study. Comorbid generic HRQoL data was the main point of interest. Tabular aggregation of homogenous and sorted data for single comorbidities was attempted but failed due to heterogeneity issues among studies. Thus, study-per-study subsumption based on utilized HRQoL instrument was incorporated. They were separated into instruments valuated by patients (e.g. EQ-5D-VAS only), instruments valuated by population (e.g. EQ-5D-5L and 15D) and measures with pre-defined summary score (e.g. SF-36, SF-12). This improves clarity for different stakeholders. Medical doctors, for example, will prefer patient valuated results due to their relevance for clinical practice. In order to reduce possible bias [37] of this review, non-significant associations of comorbidities and HRQoL were also reported. The discussion part is structured based on comorbidity.

## Results

Of the 25 studies, 16 were published in 2010 or later, while 6 were from 2014 alone. Study origin was diverse, including countries from different parts of the world. The mostly used generic instruments for measuring HRQoL were the EQ-5D in seven studies [38, 33, 39–43], the SF-12 in six studies [44–49] and SF-36 in six studies [34, 35, 50–53] as well. Table 1A shows a summary of basic study characteristics, COPD severity and comorbidity impact based on HRQoL valued by patients. Table 1B shows summary measures utilizing population valuation and Table 1C shows respective parameters for studies using pre-defined summary measure instruments.

**Table 1:** Studies on comorbidity impact.

**Part a. HRQoL instruments based on patient valuation**								
**Study**	**Country**	**Gender (%)**		**Prevalence of comorbidities in**			**Results**	
	**Sample size**	**Mean age (± SD)z**		**COPD patients and controls (if available)**				
	**Source**	**Severity of COPD**						
	**HRQoL instrument, valuation, analysis type**							
**Boros et al. 2012 [38]**	- Poland	- N(C)	8,537	- Self-report			Linear regression influence of significant predictorson health state (measured by VAS):	
	- Survey (patients across Poland)	- Female	36%	- Heart failure	21.7%			
		- Age	64.41 ± 9.86	- Ischemic heart disease	19.9%		- Heart failure: β = -0.313	
	- EQ-5D-VAS (regression of single CDs)	- GOLD Stage I	15.7%				- Other cardiovascular: β = -0.026	
		- GOLD Stage II	53.9%	- Cardiac arrhythmias	8.6%		- Endocrine/diabetes: β = -0.029	
		- GOLD Stage III	26.5%	- Other cardiovascular	32.1%		- Number of comorbidities: β = -0.139	
		- GOLD Stage IV	3.8%	- Endocrine disorders	10.2%			
				- Alimentary tract disorders	12.3%			
**Frei et al. 2014 [39]**	- Switzerland, Netherlands	- N(C)	408	- Assessment by study nurse or physician interviews and verified by medication usage			Coefficients of predictors for outcome (FT):	
		- Female	42.9%				- Depression (HADS≥11): -9.00 (-13.52, -4.48)	
	- Survey (primary care patients fromICE COLD ERIC)	- Age	67.3 ± 10.0				- Anxiety (HADS≥11): -5.53, (-10.25, -0.81)	
		- GOLD Group A	41.9%				- Peripheral artery disease: -5.02, (-10.64, 0.60)	
		- GOLD Group B	22.1%				- Cerebrovascular disease: -4.57 (-9.43, 0.29)	
	- EQ-5D-VAS (regression ofsingle CDs)	- GOLD Group C	13.5%	- Hypertension	42.2%		- Symptomatic heart disease: -3.81 (-7.23, -0.39)	
		- GOLD Group D	22.6%	- Arthrosis	29.4%			
				- Obesity	20.3%			
				- Symptomatic heart disease	20.3%			
**Cleland et al. 2007 [33]**	- UK	- N(C)	110	- Self-report			Spearman’s rank correlation (rho) between EQ-5D_VAS_ and HADS anxiety: -0.49 (p<0.001)	
	- Survey (three neighbouring practices in Aberdeen)	Female	48.2%	- Depression (HADS≥11)	20.8%			
		- Age	66.76 ± 9.60				Spearman’s rank correlation (rho) between EQ-5D_VAS_ and HADS depression: -0.54 (p<0.001)	
		- GOLD Stage I	25.5%	- Anxiety(HADS≥11)	32.7%			
		- GOLD Stage II	56.4%					
	- EQ-5D-VAS (rank correlation)	- GOLD Stage III	13.6%					
		- GOLD Stage IV	4.5%					
**Part b. HRQoL instruments based on population valuation**								
**Koskela et al. 2014 [54]**	- Finland	- N(C-15D)	731	- Medical records 15D group:			Adjusted ORs for risk factors of low 15D score (≤0.65):	
	- Pulmonary clinicsof Helsinki and Turku University Hospital	- Female	36%					
		- Age	64 ± 7	- Diabetes	111 (y)	620 (n)	- Diabetes: 2.12 (p = 0.03)	
		- FEV_1_: > 80% pred.	12.9%	- Cardiovascular	205 (y)	526 (n)	- Cardiovascular disease: 1.69 (p = 0.09)	
		- FEV_1_: 65–80% pred.	25.2%	- Hypertension	297 (y)	434 (n)		
	- 15D (backwards stepwise multivariate regression)	- FEV_1_: 40–64% pred.	43.5%	- Atrial Fibrillation	30 (y)	468 (n)	- Psychiatric disease: 4.65 (p<0.001)	
		- FEV_1_: < 40% pred.	18.5%	- Cancer	44 (y)	687 (n)	- Alcohol abuse: 2.33 (p = 0.007)	
				- Psychiatric conditions	237 (y)	488 (n)	- Hypertension (not significant)	
				- Alcohol abuse	110 (y)	621 (n)	- Cancer (not significant)	
							Attrial fibrillation not tested due to small sample size.	
**Naberan et al. 2012 [40]**	- Spain	- N(C)	4,552	- Face-to-Face interview			Correlation between EQ-5D scores and patient variables by Pearson’s r:	
	- INSEPOC (pulmonologists and family doctors)	- Female	16.7%					
		- Age	67.1±10	- CCI (mean)	1.8±1.5		- CCI: -0.330	
		- FEV_1_: % pred.	48.3±21				- HADS anxiety (HADS≥11): -0.602	
							- HADS depression (HADS≥11): -0.674	
		- EQ-5D (TTO; - logistic - regression)						
**Sundh et al. 2015 [42]**	- Sweden	- N(C)	373	- Physician interview			Association between HRQoL response and comorbidity:	
	- Secondary care respiratory units	- Female	55.8%	- Cardiovascular	59.8%			
		- Age (female)	70.5±7.58	- Diabetes	10.7%		- Musculoskeletal disease: -0.08 (p = 0.006)	
	- EQ-5D (TTO; multiple linear regression)	- Age (male)	72.2±8.11	- Musculoskeletal	24.1%		[Index]	
		- GOLD stage III	69.4%	- Osteoporosis	27.6%		- Depression (interview): -0.10 (p = 0.002)	
		- GOLD stage IV	30.6%	- Depression (interview)	16.6%		[Index]	
							- Osteoporosis: -4.65 (p = 0.049) [VAS]	
**Miravitlles et al. 2014 [43]**	- Spain	- N(C)	713	- Self-report			OR for EQ-5D utility association^5^ ^)^ with depression:	
	- DEPREPOC (multicenter)	- Female:	17%	- CCI (mean)	1.4±1.4			
		- Age	68.3±9.3	- Depression (mild to severe: BDI≥5)	74.6%		Univariate: 0.92 (p<0.05)	
	- EQ-5D (TTO)	- FEV_1_	52.1±17.3%				Multivariate: 0.94 (p<0.05)	
				- Depression (severe: BDI≥15)	14.2%		OR for EQ-5D utility association^5^ ^)^ with severedepression:	
							Univariate: 0.86 (p<0.05)	
							Multivariate: 0.90 (p<0.05)	
**Rutten-van Mölken et al. 2006 [41]**	- 13 countries	- N(C)	1,235	- Diagnosis questionnaire^4^ ^)^			Higher number of comorbidities and higher	
	- UPLIFT trial	- Female	27%				CCI score were not associated with worse	
	- EQ-5D (TTO; multivariate linear regression)	- Age	64.5±8.4	- Patients with CD	85.7%		EQ-5D VAS score. The impact of number of CDs on EQ-5D utility was highly significant (p<0.001) but small (coefficient around -0.01).	
		- GOLD Stage II	50.7%	- CCI (mean)	0.51			
		- GOLD Stage III	41.8%	- Vascular	48%			
		- GOLD Stage IV	7.4%	- Musculoskeletal	34%			
				- Metabolic	32%			
				- GI	26%			
				- Cardiac	25%			
**Blinderman et al. 2009 [55]**	- USA	- N(C)	100	- Medical records			Univariate regression r (correlation):	
	- Outpatient practices	- Female	53%	- Myocardial infarct	15%		- CCI: -0.05 (p = 0.62)	
		- Age	62.2±10.5	- Cancer	14%			
	- Documented FEV_1_<30%	- FEV_1_ (mean)	24.4±3.9	- Ulcer disease	9%			
				- Stroke	7%			
	- MILQ (univariate correlation)			- Diabetes	6%			
				- CCI (median)	1			
**Part c. HRQoL instruments with pre-defined summary measures**								
**Miguel-Diez et al. 2010 [47]**	- Spain	- N(C)	7,620	- Self-report			The presence of heart disease in patients with COPD was associated with worse scores for the physical and mental component of the SF-12.	
	- EPIDEPOC (primary care setting)	- N(C+HD)	1,770	- Blood hypertension	40.8% | 64.3%			
		- Female(C)	25%	- Hypercholesterolemia	37.7% | 44.5%			
		- Female(C+HD)	21.1%	- Diabetes	12.2% | 29.5%			
	- SF-12 (multivariate logistic regression)	- Age(C)	± 9.56	- Gastroduodenal ulcer	13.7% | 19.2%			
		- Age(C+HD)	± 8.29	- Depression	10.9% | 16.3%			
		- FEV_1_: 60–80% pred.	37.7% | 24.4%	- Anxiety	19.8% | 25.9%			
		- FEV_1_: 40–59% pred.	53.3% | 53.3%					
		- FEV_1_: <40% pred.	8.9% | 22.3%					
**Putcha et al. 2013 [56]**	- USA	- N(<3 CDs)	232	- Self-report			OR for worse health status with all independently associated CDs (adjusted for age, gender and race):	
	- NHANES (non-institutionalized population 15 counties)	- N(≥3 CDs)	611	- Selected comorbidities:				
		- Female (<3 CD)	52.1%					
		- Female (≥3 CD)	55.5%	- Prostate disease	63.6%		- CHF: 3.07 (p<0.001)	
		- Age (<3 CD | ≥3CD)	61.4 | 64.0	- Depressive symptoms (by medication)	42.4%		- CHD: 1.47 (p = 0.085)	
	- HRQOL-4 (linear and logistic regression)						- Arthritis: 1.67 (p = 0.012)	
		- Severity of COPD	Not stated	- CHF	15.1%		- Diabetes: 1.63 (p = 0.046)	
				- Diabetes	13.9%		- Depression: 1.39 (p = 0.155)	
				- CD count:			- Prostate disease: 1.63 (p = 0.045)	
				0 CD	4.3%		For every CD increase by one, the odds of worse self-rated health increased by 43%.	
				More than 2 CDs	83.6%			
**Janson et al. 2013 [45]**	- 17 countries	- N(C)	11,985	- Diagnosis questionnaire^4^ ^)^			PCS adjusted estimate (95% CI):	MCS adjusted estimate (95% CI):
	- BOLD Initiative	Female^1^ ^)^	41.0%- 54.0%					
	- SF-12 (regressionof single CDs)	Age^1^ ^)^	55.2 ± 10.8 to 64.9 ± 12.2	- Heart disease	12.6–22.9% ^1^ ^)^		-1.5 (-2.6, -0.46)	-0.12 (-1.3, 1.1)
				- Hypertension	24.4–39.4% ^1^ ^)^		-0.23 (-1.1, 0.46)	-0.51 (-1.5, 0.46)
		- No COPD	81,1%	- Diabetes	7.2–11.7% ^1^)		-2.0 (-3.6, -0.53)	-0.69 (-2.4, 1.0)
		- GOLD Stage I	8,6%	- Stroke	2.6–5.7% ^1^ ^)^		-3.0 (-5.1, -1.0)	+0.82 (-1.4, 3.1)
		- GOLD Stage II	7,9%					
		- GOLD Stage III	2,1%					
		- GOLD Stage IV	0,3%					
**Van Manen et al. 2001 [53]**	- Netherlands	- N(C)	163	- Self-report			Presence of three or more CDs was stronglyrelated to all domains of HRQoL, while the respective eight most common individual CDs (locomotive disease, hypertension, heart disease, insomnia, gastric ulcus, sinusitis, cancer, dizziness), except insomnia, were not.	
	- 28 general practices	- Female	28.2%	- Comorbidity³ ^)^	72.3%			
		- Age	66.8±9.8	- Locomotive disease	37.9%			
	- SF-36 (linear regression)	- FEV1: <50% pred.	36.8%	- Hypertension	20.1%			
		- FEV1: 50–70% pred.	39.9%	- Heart disease	15.5%			
				- Insomnia	12.3%			
		- FEV1: 70–80% pred.	23.3%	- Ulcer	9.8%			
**Wacker et al. 2014 [44]**	- Germany	- N(C)	101	- Self-report			Linear mixed models showed a negative association for heart failure (-4.9 points), myocardial infarct (-3.3), stroke (-5.6), cancer (-3.2), diabetes (-1.7) regarding PCS-but only a significant negative associationfor heart failure (-2.8), stroke (-4.0) and diabetes (-2.1) regarding MCS-12.	
	- KORA	- N(NoC)	1,220	- Cancer	4.95% | 4.7%			
	- SF-12 (linear mixed regression models)	- Female (C) | (NoC)	54,5% | 53.1%	- Diabetes	1.98% | 3.9%			
		- Age	| 51.6	- Myocardial infarction	0% | 1.8%			
		- GOLD stage I	60%	- Heart failure	1.98% | 1.1%			
		- GOLD stage II	40%	- Stroke	0.99% | 1.1%			
		- GOLD stage III+IV	1%					
**Kil et al. 2010 [34]**	- South Korea	- N(C)	91	- Self-report based on			Depressed patients had significantly (p < 0.05) lower scores in the four following dimensions:	
	- Korea University Ansan Hospital	- Female	14.3%	BDI				
		- Age	± 8.2	- Depression (BDI≥16)	15.4%			
	- SF-36 (unadjustedcomparison of group means)	- GOLD Stage I	14.2%				- Physical functioning	
		- GOLD Stage II	51.7%				- Bodily pain	
		- GOLD Stage III	29.7%				- Vitality	
		- GOLD Stage IV	4.4%				- General Health	
							Results for the other dimensions were insignificant	
**Ng et al. 2009 [48]**	- Singapore	- N(C)	189	- Self-report and verification by drug package			Adjusted OR for association of depressive symptoms with self-rated health among patients with COPD (adjusted for COPD severity, gender, age, education, smoking, comorbidity, BADL disability, dyspnea):	
	- SLAS (door-to-door census)	- N(NoC)	2,213					
		- Female(C)	64.6%					
	- SF-12 (multivariate regression)	- Female(NoC)	63.2%	- Depressive symptoms	22.8% | 12.4%			
		- Age(C | NoC)		(GDS≥5)				
		55–64	37.6% | 50.5%	- Comorbidities:			- SF-12 PCS lowest tertile: 2.35 (p = 0.041)	
		65–75	44.4% | 37.5%	None	4.2% | 7.5%		- SF-12 MCS lowest tertile: 4.17 (p = 0.001)	
		≥75	18.0% | 12.0%	1–2	52.9% | 60.0%			
		- FEV_1_: ≥80% pred.	56.1%	3 or more	42.9% | 32.5%			
		- FEV_1_: 50–80% pred.	34.4%					
		- FEV_1_: <50% pred.	9.5%					
**Bentsen et al. 2014 [57]**	- Norway	- N(C)	100	- Self-report			Unstandardizied betas for association between parameter and generic QoL:	
	- Outpatient clinic	- Female	49	- No. of comorbidities	1.67			
	- QOLS (multiple linear regression)	- Age	66.1±18.3	- Anxiety (HADS≥8)	5.9±3.9		- No. of comorbidities -0.466 (p<0.581)	
		- GOLD Stage I	0	- Depression (HADS≥8)	4.5±3.7		- Anxiety -0.320 (p<0.381)	
		- GOLD Stage II	44				- Depression -2.200 (p<0.001)	
		- GOLD Stage III	43					
		- GOLD Stage IV	13					
**Cully et al. 2006 [52]**	- USA	- N(C)	179	- Self-report			Subscales (significant factors associated with worse health status): Physical functioning (BAI, comorbidity); Role Physical (BDI), Bodily Pain (BAI); General Health (BAI), Vitality (BDI, BAI); Social Functioning (BAI, BDI); Role-Emotional (BAI, BDI); Mental Health (BAI,BDI)	
	- Veterans Medical Center	- Female	5%	- BAI (≥16)	24.6±9.3			
		- Age	65.8±10.5	- BDI (≥20)	22.5±9.4			
	- SF-36 (multiple linear regression)	- Moderate to severe	11.2%	- Comorbidities (mean)	2.4			
		- FEV_1_(mean)	45.5%					
**Krishnan et al. 2006 [35]**	- USA	- N(C)	495	- Diagnosis			Multiple linear regression coefficients for PFS and PCS (adjusted for baseline characteristics):	
	- Routine data Erie and Niagara Counties, NY	- Female	45.2%	questionnaire				
		- Age	64.15±9.97	- Anemia	7.47%			
		- GOLD Stage I	0.6%	- Myocardial Infarction	11.31%		- β (PFS_Diabetes) = -0.13 (p<0.0001)	
	- SF-36 (multiple linear regression)	- GOLD Stage II	87.7%	- Renal Disease	2.02%		- β (PCS_Diabetes) = -0.07 (p = 0.02)	
		- GOLD Stage III	11.1%	- Diabetes	16.57%		Causal relationship for anemia and HRQoL not established. History of myocardial infarct and renal disease not significant.	
		- GOLD Stage IV	1.2%					
**Rascon-Aguilar 2011 [51]**	- USA	- N(C)	86	- Self-report			Comparison of SF-36 means between COPD+GERD and COPD only:	
	- Pulmonary clinic University of Florida/Jacksonv.	- Female(GERD)	41%	- Hypertension	43.8 | 47.1			
		- Female(nGERD)	46.3%	- Coronary artery disease	28.1 | 9.4		Bodily pain: 51.7 | 66.7 (p<0.02)	
		- Age(GERD)	66.0±9.9				Mental health: 60.5 | 71.3 (p<0.03)	
	- SF-36 (comparison of means)	- Age(nGERD)	68.8±7.0	- Arthritis	25.0 | 30.19		PCS summary score: 29.3 | 33.8 (p<0.05)	
		- FEV_1_(GERD) pred.	45.9%±16%	- Hypercholesterolemia	21.8 | 15.09		Remaining scales and scores not significant.	
		- FEV_1_(nGERD) pred.	40.7%±17.6%	- Diabetes	12.5 | 13.21			
				- Depression	12.5 | 13.21			
**Xiang et al. 2014 [49]**	- Hong Kong	- N(C)	142	- Face-to-Face interview			MCS and PCS scores were not significantly associated with number of illnesses but werea significant predictor for SGRQ QoL.	
	- 1 hospital	- N(Controls)	218					
	- COPD medical records	- Female(C)	16.9	- GDS(C)	4.7±4.1			
		- Female(Control)	24.8	- GDS(Control)	2.8±3.1			
	- SF-12 (multiple linear regression)	- Age(C)	73.9±6.2	- No. of comorbidities (C)	3.0±1.7			
		- Age(Control)	75.0±6.0					
		- GOLD Stage I	6.3%	- No. of comorbidities (Control)	1.9±1.3			
		- GOLD Stage II	15.5%					
		- GOLD Stage III	45.8%					
		- GOLD Stage IV	32.4%					
**Lopez Varela et al. 2013 [46]**	- Latin America	- N(C)	759	- Self-report			An association between increased comorbidity score (unweighted) and deteriorating general health status was observed. Of the evaluated comorbidities, diabetes had the strongest effect on HRQoL detoriation.	
	- Multi-stage cluster sampling in five Latin American cities	- N(NoC)	4,555	- Heart disease	13.7%| 12.7%			
		- Female(C)	47.7%	- Hypertension	37.2% | 33.7%			
		- Female(NoC)	62.6%	- CVA	3.2% | 2.1%			
		- Age(C)≥60	33.5%	- Cardiovascular	41.5% | 38.8%			
		- Age(NoC)≥60	28.7%	- Diabetes	8.4% | 9.9%			
	- SF-12 (comparison of means)	- FEV_1_: <70% pred.	14.3%	- Peptic ulcer	31.8% | 29.9%			
				- Lung cancer	1.1% | 0.1%			
				- Asthma	22.8% | 10.5%			
**Van Manen et al. 2003 [50]**	- Netherlands	- N(C)	148	- Self-report			Linear regression influence of dichotomous comorbidity on SF-36 score (p<0.05):	
	- General practices	- N(NoC)	364	- 1–2 chronic diseases	52.7% | 51,9%			
	- SF-36 (dichotomous linear regression)	- Female(C)	30.4%	- 3–4 chronic diseases	15.5% | 8,8%		Physical functioning: β = -12	
		- Female(NoC)	57.7%	- ≥5 chronic diseases	4.1% | 2,2%		Role functioning physical: β = -24.7	
		- Age(C) ≥60	77.03%				Social functioning: β = -10.8	
		- Age(NoC) ≥60	69.51%				Mental health: β = -8.0	
		- FEV_1_: <50% pred.	37.2%				Role functioning emotional: β = -16.2	
		- FEV_1_: 50–70% pred.	38.5%				Vitality: β = -14.0	
							Bodily pain: β = -16.3	
		- FEV_1_: 70–80% pred.	24.3%				General health: β = -13.0	
**Shavro et al. 2012 [58]**	- India	- N(C)	58	- Unknown			No association between comorbid illness and HRQoL. [Unknown cause. Small sample size? Aspects of Indian culture?]	
	- 1 hospital	- Female	7%	- Gastric disease	21%			
	- WHOQOL-BREF	- Age	62.4±7.8	- Hypertension	19%			
		- GOLD Stage I	1.7%	- Diabetes Mellitus	17%			
		- GOLD Stage II	79.3%	- Heart disease	16%			
		- GOLD Stage III	19%	- 0 comorbidities	34%			
				- 1 comorbidity	29%			
				- 2 comorbidities	24%			
				- ≥3 comorbidities	12%			
**Wijnhoven et al. 2003 [59]**	- Netherlands	- N(C)	161	- Face-to-Face interview			Adjusted significant ORs for poor HRQoL (NHP total score):	
	- General practices	- Female	44.7%					
	- NHP (logistic - regression)	- Age	61.0±10.3	- No CD	46.6%		- >1 CD: 3.22	
		- FEV_1_: % pred.	60.7±15.0	- One CD	30.4%		- Presence of musculoskeletal disorders: 2.52	
				- More than one CD	23.0%			
				- Musculoskeletal	27.3%		Not significant: 1 CD; Cardiac disease; Hypertension	
				- Cardiac	19.3%			
				- Hypertension	17.4%	

^1)^: depending on COPD status and severity grade

^3)^: The presence of comorbidity was only calculated for patients who filled out the respective questionnaire

^4)^: Patients were asked if they had a physician diagnosis for respective comorbidities

^5)^: OR<1 implicates lower chance for depression when EQ-5D score increases; BAI: Beck Anxiety Inventory; BDI: Beck Depression Inventory; C: COPD; CCI: Charlson comorbidity index; CD: Comorbid disease; CHF: Congestive heart failure; CHR: Coronary heart disease; ERS: European Respiratory Society; GERD: Gastroesophageal reflux disease; GOLD: Global Initiative for Chronic Obstructive Lung Disease; HADS: Hospital Anxiety and Depression Scale; HD: Heart Disease; MCS: Mental component summary score; MILQ: Multidimensional Index of Life Quality; NHP: Nottingham Health Profile; OR: Odds ratio; PCS: Physical component summary score; PFS: Physical Functioning Scale; PMR: Patient medical record; Post-BD: post-bronchodilator; SGRQ: St George's Respiratory Questionnaire; TTO: Time-trade-off

All but one EQ-5D study reported using the EQ-5D-3L version with a 3-level distinction of problems reported. Miravitlles et al. 2014 [43] did neither report the used version, nor the used value sets. Three studies used patient’s VAS valuation [33, 39, 38]. Three studies used value sets based on time-trade-off valuations by a general population, taken from a UK [60], US [61] and Spanish [62] setting. Instruments used by a single study each are the self-administered 15-dimension 15D [32], the 35-item Multidimensional Index of Life Quality (MILQ) [63], the 16-item Quality of Life Scale (QOLS) [64], the 26-item World Health Organization Quality of Life-BREF (WHOQOL-BREF) [65], the 4-item Health Related Quality of Life-4 (HRQOL-4) [66] and the 38-item Nottingham Health Profile (NHP) [67]. They were utilized by one study [54, 56, 59, 58, 57, 43, 55] each. Valuation of the 15D instrument was done by population-based multiattributive utility theory [32]. Besides EQ-5D and 15D, no index instruments were used. References regarding the validation of the used instrument for COPD as well as respective comorbidity were only given by one study [51]. Sample sizes ranged from 58 [58] to 11,985 [45] COPD patients. The prevailing gender was male in the majority of studies and was even as high as 95% in a study [52] with veterans and 93% in a study [58] from India. The average age was above 60 years in all studies respectively. The severity of COPD was assessed in nearly all studies. Classification of COPD severity was mostly based on GOLD criteria but other cut-off points for predicted forced expiratory volume in 1 second (FEV1) were also used for classification of patients. Some studies only stated average FEV1 values and one study [56] did not state severity classification at all. The majority of patients had an average FEV1 predicted of around and/or above 50% but heterogeneity was high. Studies [42, 55, 49] with mainly severe to very severe cases of COPD were also present. Seven studies [50, 48, 56, 52, 49, 57, 58] assessed the number of comorbidities per patient. The majority of patients seem to be afflicted by around two or more comorbidities. All studies reported some form of association between specific comorbidity and worse HRQoL. However, the comorbid influence as well as its significance differed among studies. The most prevalent evaluated diseases were cardiovascular disease (CVD), which is a far reaching umbrella term for diseases of the heart and/or blood vessels, as well as depression and anxiety. Ten studies [38, 39, 45, 54, 47, 56, 44, 59, 53, 46] looked at the influence of cardiovascular disease on HRQoL. A negative association was stated in seven of the studies, while three studies [54, 59, 53] did not find a significant association. The non-significance was mentioned by the authors but not explained through specific reasons. Regarding depression, only one [56] of the ten studies [33, 39, 34, 40, 48, 56, 42, 43, 57, 54] did not find a significant negative association with HRQoL. The EQ-5D index, based on TTO, was associated with depression in three studies [40, 42, 43]. In another study [54], psychiatric disease had an adjusted odds ratio (OR) of 4.65 for low 15D score. Ng et al. 2009 [48] calculated an adjusted OR of 4.17 for depression and low self-rated health measured by SF-12. Two additional studies [52, 57] report a significant negative association for depression and HRQoL measured by SF-36 and QOLS. A comparable picture emerges for anxiety, which had a non-significant association in only one study [57]. Diabetes was associated with worse HRQoL in all respective studies [38, 45, 54, 35, 46, 56, 44]. Two studies [59, 42], using EQ-5D index and NHP, also found a negative influence of musculoskeletal disease on HRQoL, the former study stating a value of -0.08 (p = 0.006) based on multiple linear regression for the association between presence of musculoskeletal disease and EQ-5D index. The presence of comorbidity, irrespective of type, was also associated with lower HRQoL scores in six [46, 56, 59, 50, 40, 53] out of ten studies and was significantly associated with worse physical functioning in one study [52]. Presence of more than one comorbidity resulted in an adjusted OR of 3.22 for poor HRQoL, measured by NHP [59]. Rutten-van Mölken et al. 2006 [41] stated, that a higher number of comorbidities and higher Charlson Comorbidity Index (CCI) score was not associated with lower EQ-5D-VAS score, while the impact on EQ-5D index was significant but only small. Blindermann et al. 2009 also found CCI not to be associated with worse MILQ scores but considered this to be rooted in the low CCI median of 1 they started with. Three studies [49, 58, 57] did not find any significant association for number of comorbidities and worse generic HRQoL. Other significant negative associations were found for insomnia [53], alcohol abuse [54], arthritis [56], gastroesophageal reflux disease (GERD) [51] and osteoporosis [42].

## Discussion

The results clearly show that specific concomitant diseases in COPD were associated with worse generic HRQoL, irrespective of utilized instrument. However, the degree of HRQoL impact varied and some studies delivered contradicting results.

### Comorbid CVD

One of the comorbidities with significant influence on HRQoL was CVD. Boros et al. 2012 [38] calculated a standardized linear regression coefficient of -0.313 for the association of the EQ-5D-VAS and presence of heart failure in COPD. This transforms into a 15 point reduction on the VAS scale (according to author correspondence). This standardized coefficient is around 10 times higher than standardized EQ-5D-VAS coefficients for other cardiovascular diseases excluding ischemic heart disease (-0.145), in the same study. Frei et al. 2014 [39] stated a EQ-5D-VAS predictor of -4.6 and -3.8 for cerebrovascular and symptomatic heart disease respectively, while Wacker et al. 2014 [44] found a significant negative association for heart failure as well as stroke and the physical and mental component summary among patients with COPD. This receives additional importance because observational data indicates, that COPD patients are at increased risk for developing CVD [68]. Three [59, 54, 53] out of ten respective studies [38, 53, 39, 54, 45, 47, 44, 56, 59, 46] did not find a significant association between comorbid CVD and HRQoL. Van Manen et al. 2001 [53] explain the lack of significant association for heart disease by pointing to the relative low number of patients with the disease (n = 25) in their study. Koskela et al. 2014 [54] did not state a reason for the lack of association and Wijnhoven et al. 2003 [59] only found a negative association for heart disease and asthma but not for COPD. They point towards differences in disease characteristics as possible explanation. In addition to this, CVD is a far reaching umbrella term for diseases of the heart and circulation and this may explain inconsistencies among results since different patient populations may be affected by different cardiovascular disorders and different severity grades. This general limitation is mentioned by Sundh et al. 2015 [42].

### Comorbid depression and anxiety

Other comorbidities with strong association for worse HRQoL were depression and anxiety. 11 studies evaluated its comorbid influence on HRQoL. Cut-off points for being depressed were ≥ 11 for the HADS in three studies. Interestingly, the only study [57] using a lower HADS cut-off point of ≥8 stated a non-significant (p<0.381) HRQoL association for anxiety but not depression. The non-significance may be explained by the lower cut-off point and hence, a lower severity grade of overall anxiety in this patient population. This is confirmed by looking at the HADS-A mean scores. While Bentsen et al. [57] stated a HADS-A mean of 5.9 (SD: 3.9), the other two studies [39] stated higher means of 9 (SD = 4.2) for females, 7.2 (SD: 4) for males or an average of 7 (SD: na). Table 2 shows an overview of respective results among studies using the EQ-5D instrument. It became apparent that depression ranked first among comorbidities with significant association for worse HRQoL in all four studies. Consequentially, in all three studies [33, 39, 40] where a comparison was possible, depression had always a stronger influence on worse HRQoL than anxiety. Naberan et al. 2012 [40] calculated an r value of -0.674 (-0.602) for the correlation of HADS depression (anxiety) and the EQ-5D index score and found this to be the best correlation in their study. In another study [39], depression was associated with a reduction of EQ-5D-VAS score by around 9 points. Interesting from a practical perspective, Cleland et al. 2007 [33] stated the possible use of the EQ-5D-VAS as quick and easy screening tool for patient’s mental health in COPD. This procedure would be supported by the results of Frei et al. 2014 [39], who also found a strong association for depression and low EQ-5D-VAS score. Irrespective of these two studies, other authors point towards usage of health status measures as indicators for depression in COPD as well [69]. A problem regarding evaluation of index based influence of comorbidity on HRQoL in COPD is that depression and anxiety, two disorders which showed a strong influence on HRQoL, are completely missing from indices like the CCI. Therefore exclusively using these indices will likely fail to deliver a complete picture of comorbid associations with HRQoL. In general, we would therefore agree with Frei et al. 2014 [39], who stated, that comorbidity based indices which predict mortality are not designed to evaluate HRQoL status. They may serve as indicator but attention has to be paid when evaluating their results.

**Table 2:** Results and ranks of depression and anxiety for comorbid influences on HRQoL by studies using EQ-5D.

	Result type	Result D	Result A	Rank D	Rank A	Total range
Cleland et al. 2007 [33]	Spearman’s rho EQ-5D-VAS	-0.54	-0.49	1 (2)	2 (2)	-0.49 to -0.54
Frei et al. 2014 [39]	Regression coefficient EQ-5D-VAS	-9.00	-5.53	1 (5)	2 (5)	-3.81 to -9.00
Naberan et al. 2012 [40]	Pearson’s r EQ-5D-Index	-0.67	-0.60	1 (3)	2 (3)	-0.33 to 0.67
Sundh et al. 2015 [42]	Regression coefficient EQ-5D-Index	-0.10	n.a.	1 (3)	n.a.	-0.07 to -0.10

Total range refers to the range of results among comorbidities with significant influence on HRQoL in the respective study

D: depression; A: anxiety; (): number of available ranks

### Comorbid diabetes

Diabetes was associated with worse HRQoL in all respective studies and among many different instruments including EQ-5D-VAS [38], SF-12 [45, 46, 44], SF-36 [35], 15D [54] and HRQOL-4 [56]. In addition to this finding, comorbid diabetes seems to worsen the prognosis [70] and lengthen the hospital stays of COPD patients with acute exacerbations due to immune dysfunction [71].

### Comorbid musculoskeletal disease

Musculoskeletal disease also was significantly associated with worse HRQoL in two studies [42, 59]. The association for presence of the disease and EQ-5D index score was -0.08 (p = 0.006) [42]. Interestingly, muscle wasting in COPD seems also to be a better predictor for mortality, than BMI [72, 73]. Muscle wasting is connected to fatigue and reduced activity [74]. Since mobility, activity and self-care are three dimensions of the EQ-5D a drop of HRQoL scores in patients who suffer from muscle wasting is not very surprising.

### Number of comorbidities

Three [57, 49, 58] out of eleven studies [38, 41, 52, 53, 50, 46, 57, 58, 56, 59, 49] did not find a significant association between number of comorbidities and worse generic HRQoL. Shavro et al. 2012 [58] mention this finding to be surprising and possible rooted in the small sample size (n = 58) and/or aspects of Indian culture. A connection between comorbidities and worse scores for the WHOQOL-BREF or SGRQ scores was not found in their study. Xiang et al. 2014 [49] found number of comorbidities to be associated with worse SGRQ results but not with SF-12. Bentsen et al. 2014 [57] found the same relation for QOLS and SF-12. It seems counter-intuitive that disease specific instruments react more sensitive to the presence of comorbidity compared to generic instruments, unless the respective comorbidity has a significant effect on COPD symptoms. Unfortunately, since only number of comorbidities were evaluated by both studies, specific conclusions for individual diseases can’t be drawn and the statistical power is low to begin with. However, advantages of using disease-specific and generic instruments together have been stated before [75]. Conducting a review on the comorbid influences on HRQoL measured by disease-specific instruments, would thus be interesting from a research perspective.

### Comparing comorbid costs and HRQoL

When considering the effect of comorbidities on the cost-effectiveness of COPD intervention, next to HRQoL, the cost impact is relevant. A previous systematic review found comorbidities in COPD patients to be associated with significant excess cost [76]. However, the impact of comorbidities on cost of COPD patients cannot directly be compared with that of HRQoL: The instruments used for measurement of HRQoL were diverse, as were statistical measures of comorbidity impact. Furthermore, control groups were lacking in many HRQoL studies. In contrast, most of the HRQoL studies measured stage of COPD while this information was sparse in cost studies. A simultaneous view upon cost and HRQoL impact of comorbidities is thus hindered by possible differences in study patients, and by diverging methods. Simultaneous study of both dimensions in individual patients is needed to provide a comprehensive view of comorbidity impact.

### Limitations and strength of this review

Limitations of this review include the non-active search for studies which consider COPD as the comorbidity and other diseases as index disease. However, by doing so, an increase in already high study heterogeneity was likely prevented to some degree. The present heterogeneity is rooted in the evaluation of different patient populations and comorbidities but also in the use of different HRQoL instruments, different value-sets and different outcome measurements, which decrease comparability even further. Moreover, when population-based value sets are being used to aggregate HRQoL, applying an experience-based approach rather than one based on hypothetical health states as in three studies [42, 41, 40] could help to increase physician acceptance of HRQoL results, shifting the focus to actual patient experience [77]. Last but not least the severity of comorbidities was not assessed in the studies under review. Thus, the influence of comorbid severity on HRQoL remains unclear.

Strength of this review is the aggregation of generic evidence on HRQoL pertaining the comorbid influences in COPD and illustrating evidence in aggregated and comprehended form. Over 1700 studies were filtered and screened and to our knowledge this is the first review evaluating comorbid effects on generic HRQoL. Furthermore, using multiplicative methods, which showed superior performance compared to minimum or additive methods when trying to incorporate selective comorbid burden into health state utilities [78–80] could allow creating decision analytical COPD models by the aggregated data, which resemble clinical reality to a higher degree.

## Conclusion

Comorbidities in COPD are significantly associated with worse HRQoL among all used instruments. The majority of evidence was generated for CVD, depression and anxiety as well as diabetes but other comorbid conditions like musculoskeletal disease, have a worsening influence on HRQoL in COPD as well. The sole presence of quantitative comorbidity was also connected to lower HRQoL. These results should be considered in clinical practice and in studies evaluating interventions in respective patient populations. Not considering the HRQoL impact of existing comorbidity might lead to inappropriate clinical management and to biases in evaluation studies. It became apparent that facilitating multimorbid intervention guidance, instead of applying a parsimony based single disease paradigm, constitutes an important current and future goal for patient management in COPD.

## Supporting Information

S1 File PRISMA checklist. (PDF)

S2 File Excluded studies and reasons. (DOC)
