# Supplementary material for: Comorbid Influences on Generic Health-Related Quality of Life in COPD: A Systematic Review
Source: PLoS One. 2015 Jul 13;10(7):e0132670. doi: 10.1371/journal.pone.0132670 (PMC4500578; doi:10.1371/journal.pone.0132670)
Supplement: S2 File — (DOC) [file pone.0132670.s002.doc]

**Removed studies and respective reason:**

Putcha N, Han MK, Martinez CH, Foreman MG, Anzueto AR, et al. (2014) Comorbidities of COPD have a major impact on clinical outcomes, particularly in African Americans. Chronic Obstr Pulm Dis (Miami) 1: 105-114.

**Reason:** No generic instrument.

Urff M, van den Berg JW, Uil SM, Chavannes NH and Damoiseaux RA (2014) Depression and heart failure associated with clinical COPD questionnaire outcome in primary care COPD patients: a cross-sectional study. NPJ primary care respiratory medicine 24: 14066.

**Reason:** No generic instrument.

Weldam SW, Lammers JW, Heijmans MJ and Schuurmans MJ (2014) Perceived quality of life in chronic obstructive pulmonary disease patients: a cross-sectional study in primary care on the role of illness perceptions. BMC family practice 15: 140.

**Reason:** No generic instrument.

Black-Shinn JL, Kinney GL, Wise AL, Regan EA, Make B, et al. (2014) Cardiovascular disease is associated with COPD severity and reduced functional status and quality of life. Copd 11: 546-551.

**Reason:** No generic instrument.

Ohayon MM (2014) Chronic Obstructive Pulmonary Disease and its association with sleep and mental disorders in the general population. J Psychiatr Res 54: 79-84.

**Reason:** No generic instrument.

Raherison C, Tillie-Leblond I, Prudhomme A, Taille C, Biron E, et al. (2014) Clinical characteristics and quality of life in women with COPD: an observational study. BMC Womens Health 14: 31.

**Reason:** No generic instrument.

Caillaud D, Chanez P, Escamilla R, Burgel PR, Court-Fortune I, et al. (2014) Association of chronic nasal symptoms with dyspnoea and quality-of-life impairment in chronic obstructive pulmonary disease. Respirology 19: 346-352.

**Reason:** No generic instrument.

Cheville AL, Basford JR, Dos Santos K and Kroenke K (2014) Symptom burden and comorbidities impact the consistency of responses on patient-reported functional outcomes. Archives of physical medicine and rehabilitation 95: 79-86.

**Reason:** No generic instrument.

Burgel PR, Escamilla R, Perez T, Carre P, Caillaud D, et al. (2013) Impact of comorbidities on COPD-specific health-related quality of life. Respir Med 107: 233-241.

**Reason:** No generic instrument.

Sundh J, Stallberg B, Lisspers K, Montgomery SM and Janson C (2011) Co-morbidity, body mass index and quality of life in COPD using the Clinical COPD Questionnaire. Copd 8: 173-181.

**Reason:** No generic instrument.

Balcells E, Gea J, Ferrer J, Serra I, Orozco-Levi M, et al. (2010) Factors affecting the relationship between psychological status and quality of life in COPD patients. Health Qual Life Outcomes 8: 108.

**Reason:** No generic instrument.

Omachi TA, Katz PP, Yelin EH, Gregorich SE, Iribarren C, et al. (2009) Depression and health-related quality of life in chronic obstructive pulmonary disease. The American journal of medicine 122: 778 e779-715.

**Reason:** Inadequate coverage of comorbid influence on HRQoL.

Heyworth IT, Hazell ML, Linehan MF and Frank TL (2009) How do common chronic conditions affect health-related quality of life? The British journal of general practice : the journal of the Royal College of General Practitioners 59: e353-358.

**Reason:** Inadequate coverage of COPD.

Hynninen MJ, Pallesen S and Nordhus IH (2007) Factors affecting health status in COPD patients with co-morbid anxiety or depression. International journal of chronic obstructive pulmonary disease 2: 323-328.

**Reason:** No generic instrument.

Ng TP, Niti M, Tan WC, Cao Z, Ong KC, et al. (2007) Depressive symptoms and chronic obstructive pulmonary disease: effect on mortality, hospital readmission, symptom burden, functional status, and quality of life. Arch Intern Med 167: 60-67.

**Reason:** No generic instrument.

Yeo J, Karimova G and Bansal S (2006) Co-morbidity in older patients with COPD--its impact on health service utilisation and quality of life, a community study. Age Ageing 35: 33-37.

**Reason:** No generic instrument.

Aydin IO and Ulusahin A (2001) Depression, anxiety comorbidity, and disability in tuberculosis and chronic obstructive pulmonary disease patients: applicability of GHQ-12. General hospital psychiatry 23: 77-83.

**Reason:** No generic instrument.

Carrasco-Garrido P, de Miguel-Diez J, Rejas-Gutierrez J, Martin-Centeno A, Gobartt-Vazquez E, et al. (2009) Characteristics of chronic obstructive pulmonary disease in Spain from a gender perspective. BMC Pulmonary Medicine 9.

**Reason:** Inadequate coverage of comorbid influence on HRQoL.

Cecere LM, Littman AJ, Slatore CG, Udris EM, Bryson CL, et al. (2011) Obesity and COPD: Associated symptoms, health-related quality of life, and medication use. COPD: Journal of Chronic Obstructive Pulmonary Disease 8: 275-284.

**Reason:** No generic instrument.

Domingo-Salvany A, Lamarca R, Ferrer M, Garcia-Aymerich J, Alonso J, et al. (2002) Health-related quality of life and mortality in male patients with chronic obstructive pulmonary disease. American Journal of Respiratory and Critical Care Medicine 166: 680-685.

**Reason:** Inadequate coverage of comorbid influence on HRQoL.
